# SARS Coronavirus Detection

**DOI:** 10.3201/eid1007.030678

**Published:** 2004-07

**Authors:** Andreas Nitsche, Brunhilde Schweiger, Heinz Ellerbrok, Matthias Niedrig, Georg Pauli

**Affiliations:** * Robert Koch-Institut, Berlin, Germany

**Keywords:** coronavirus, SARS, real-time RT-PCR, molecular diagnostics

## Abstract

We developed a set of three real-time reverse transcription–polymerase chain reaction (PCR) assays that amplify three different regions of the SARS-associated coronavirus (SARS-CoV), can be run in parallel or in a single tube, and can detect <10 genome equivalents of SARS-CoV. The assays consider all currently available SARS-CoV sequences and are optimized for two prominent real-time PCR platforms.

## The Study

Recently, a new coronavirus was identified as the suspected causative agent of an increased number of atypical pneumonia cases reported from Hong Kong, Singapore, Vietnam, and Canada (1–4). Subsequent publications demonstrated that this new coronavirus was detectable in patients with severe acute respiratory syndrome (SARS) (5,6), classified according to the World Health Organization's case definition (7). During the first 6 months of 2003, a total of 8,422 patients were affected. This fact, together with the reappearance of the SARS-associated coronavirus (SARS-CoV) in China in late 2003, makes it clear that rapid and reliable diagnostic tools are essential for accurate disease reporting and subsequent disease management.

Because a defined treatment program and vaccination strategy are lacking, the main strategy to counteract the spread of this emerging virus is timely identification and isolation of infected persons. SARS patients' typical initial symptoms include fever, cough, and headache, similar to many acute viral respiratory infections. Therefore, molecular-based diagnostic methods are applied to rapidly identify SARS-CoV–infected persons. Recently, nested and real-time reverse transcription–polymerase chain reaction (RT-PCR) assays to detect SARS-CoV have been published (5,8). These assays—the first tools to detect SARS-CoV in patients with SARS—were based on the short stretches of viral sequence identified as the RNA-directed RNA polymerase of a new microbe.

Subsequently, sequences from several SARS-CoV isolates were determined, and all of these sequences were closely related, as would be expected during the clustered outbreaks in 2003. However, the genomes of RNA viruses, including those of coronaviruses, tend to vary over time and with location (9–12). Recently, the sequence variations of SARS-CoV during the first epidemic phases in China in 2003 were reported. The neutral mutation rate for SARS-CoV was almost constant and similar to that of known RNA viruses; the S protein, responsible for virus-host receptor recognition, displayed the most extensive amino acid changes (13). In addition, the sequence analysis of isolates from recent SARS patients in China in 2004 has shown that 98.8%–99.4% of the 3,768 bases of S gene, 99% of 658 bases of M gene, and 99% of 1,068 bases of N gene are isogenous with those submitted to public databases, which date back to the first epidemic in spring 2003 (14). However, even these minimal changes could render existing PCR assays ineffective should SARS-CoV reemerge (15).

To improve the ability to detect SARS-CoV safely and reduce the risk of eliciting false-negative results caused by genome sequence variations, we established three individual real-time RT-PCR assays. Target sequences were chosen by using the following criteria: 1) the regions are distributed over the whole genome, including the nonstructural polyprotein 1a and 1ab genes and the spike glycoprotein gene (Table 1); 2) the regions are highly conserved among the 89, 90, and 100 respective sequences available in public sequence databases; 3) the regions are suitable for the design of a real-time RT-PCR assay; and 4) the designed primers, 5´-nuclease probes, and amplicons displayed no considerable homology to other viruses, including human CoV OC43 and 229E in BLAST searches (available from http://www.ncbi.nlm.nih.gov/BLAST/).

**Table 1:** Primers and 5´-nuclease probes of the three SARS coronavirus–specific assays^a^

Primer/probe	Primer/probe sequence	Oligonucleotide orientation	Nucleotide position^b^	Tm (°C)^c^
	**NS pp1a (133 bp)** ^d^			
pp1a F	GCCgTAgTgTCAgTATCATCACC	S	4609–4631	56.6
pp1a R	AATAggACCAATCTCTgTAAgAgCC	A	4741–4717	56.7
pp1a TM	F-TCACTTCgTCATCAAAgACATC XT gAggAgC p	S	4661–4690	66.2
	**NS pp1ab (88 bp)^d^**			
NS F	TTTTgTTgTTTCAACTggATACCAT	S	14387–411	57.0
NS R	GAAACTgAgACgCgAgCTATgT	A	14474–453	57.3
NS TM	F-CATCCTgATTATgTACgACTCCTAAC XT CACgAA p	A	14445–413	64.4
	**Surface spike glycoprotein (79 bp)^d^**			
SS GP F	gAggTCTTTTATTgAggACTTgCTC	S	23879–903	57.1
SS GP R	gCATTCgCCATATTgCTTCAT	A	23957–937	57.3
SS GP TM	F-AAgCCAgCATCAgCgAgTgTCACCTTA XT p	A	23935–908	66.7

^a^SARS, severe acute respiratory syndrome; S, sense; A, antisense; F, 6-carboxyfluorescein attached to 5´-terminus (FAM); T, 5-carboxytetramethylrhodamine (5-TAMRA) attached to 5-ethylamino-dThymidin; NS, nonstructural; pp1ab, polyprotein 1ab gene; Tm, melting temperature; TM, TaqMan. ^b^Based on AY274119 isolate TOR2. ^c^Thermodynamic Tm. All oligonucleotides were synthesized by TIB MOLBIOL, Berlin. ^d^Amplicon length.

These assays were based on the fluorogenic oligoprobe chemistry, which uses the 5´-exonuclease activity of the DNA polymerase to generate a more specific signal than that produced by the use of SYBR Green I (8). The real-time RT-PCR assays were successfully run on the Applied Biosystems real-time PCR systems (SDS7700 and SDS7000; Applied Biosystems, Foster City, CA) as well as on the Roche LightCycler (Roche Diagnostics GmbH, Mannheim, Germany). All assays were designed as one-step RT-PCR reactions to be run under identical conditions on the respective PCR platform. This system allowed the simultaneous detection of different SARS-CoV regions in a single PCR run. Moreover, we could combine the three assays in a single tube, which might be important when clinical material is limited. Finally, the assays were compared to the 5´-nuclease assay published recently (5) and to a commercially available real-time PCR kit (Real-Art HPA-Coronavirus LC RT PCR Reagents, Artus GmbH, Hamburg, Germany).

After optimization of primer and 5´-nuclease probe concentration and annealing temperature, reaction conditions for our 5´-nuclease assay were as follows. For the RT-PCR performed on the Applied Biosystems platforms, each 25-µL reaction contained 12.5 µL of 2xQuantiTect Probe RT-PCR Master Mix (Qiagen, Hilden, Germany), 10 pmol of each primer, 3 pmol of 5´-nuclease probe, and 0.25 µL of QuantiTect Probe RT Mix. RNase-free water was added up to 23 µL, and 2 µL of RNA was used. Cycling conditions were 30 min at 50°C for RT reaction, 15 min at 95°C for inactivation of RT, activation of the Taq DNA polymerase, and cDNA denaturation, followed by 45 cycles of 15 s at 95°C and 30 s at 60°C. Total running time was 140 min.

For LightCycler RT-PCR reactions, each 20-µL reaction included 7.5 µL of 2.7xLightCycler RNA Master Hybridization Probes mix (Roche Diagnostics GmbH), 10 pmol of each primer, 3 pmol of the 5´-nuclease probe, and 1.3 µL Mn (OAc)_2_ (50 mmol/L). RNase-free water was added up to 18 µL, and 2 µL of RNA was used. Cycling conditions were 20 min at 55°C for the RT reaction, 30 s at 95°C for initial denaturation, followed by 45 cycles of 1 s at 95°C, 10 s at 55°C, and 10 s at 72°C. Total running time was 55 min. The combined assays were set up by adding all primers and probes in the same concentration; the amount of water was reduced accordingly. Protocols are also available from the Robert Koch-Institut homepage (available from www.rki.de/INFEKT/SARS/PCRPROTOCOL.PDF). The human L13 gene and the human cyclophilin gene (16) were amplified under identical reaction conditions as the SARS-CoV–specific assays on the ABI platforms and the LightCycler, respectively, to act as amplification controls.

To evaluate the sensitivity of the SARS-CoV–specific assays, RT-PCRs were performed repeatedly on serial dilutions of RNA extracted with the Viral RNA Kit (Qiagen) from cultured SARS-CoV with defined amounts of genome equivalents (GE) by using the international standard of the European Network for the Diagnostics of Imported Viral Diseases (ENIVD), distributed through the Robert Koch-Institut (available from: http://www.rki.de/INFEKT/SARS/DATASHEET.PDF). Results are shown in Table 2. Detection limits of the three new assays were <10 GE. Comparison of the threshold cycle (C_T_) values showed that the new assays were at least as sensitive as the previously described assays (5,8) and the commercially available kit. When we combined the assays in a single tube targeting three different regions on the same RNA template, the C_T_ was reduced by 1 to 2 cycles (SDS7700), which suggests either that sensitivity was unchanged or, when there was an increase, that it was attributable to the combination of all three signals (LightCycler). Subsequent agarose gel analysis during optimization steps of the PCR confirmed the simultaneous amplification of the three RT-PCR products (Figure).

**Table 2:** C_T_ values and standard deviation of serial dilutions of SARS-CoV RNA subjected to different real-time RT-PCR assays^a^

GE	Real-time RT-PCR systems	Artus kit n = 4	Drosten et al. (5) n = 6	NS pp1a^b^ n = 7	NS pp1ab^b^ n = 7	SS GP^b^ n = 7	NS pp1a/NS pp1ab/SS GP^b^ n = 6
500	LightCycler	28.69 ± 0.46	28.16 ± 0.04	29.57 ± 0.87	29.56 ± 0.18	29.47 ± 0.53	28.53 ± 0.26
	SDS7700	n.d.	27.29 ± 0.09	29.63 ± 0.94	30.92 ± 0.09	26.98 ± 0.65	26.11 ± 0.40
50	LightCycler	32.68 ± 0.26	45.00	33.85 ± 0.60	32.83 ± 0.41	32.83 ± 0.41	32.48 ± 0.69
	SDS7700	n.d.	31.62 ± 0.45	33.82 ± 0.23	34.73 ± 1.04	30.33 ± 0.26	29.29 ± 0.67
10	LightCycler	34.42 ± 0.20	45.00	36.72 ± 0.42	35.99 ± 0.55	35.33 ± 0.48	34.50 ± 0.25
	SDS7700	n.d.	38.37 ± 4.02	35.69 ± 0.35	36.94 ± 0.10	32.93 ± 0.81	32.01 ± 0.44
5	LightCycler	35.66 ± 0.40	45.00	45.00	37.61 ± 0.55	37.12 ± 0.35	37.20 ± 0.30
	SDS7700	n.d.	41.15 ± 2.17	37.31 ± 0.77	37.64 ± 0.67	34.55 ± 0.50	34.09 ± 0.24

^a^C_T_, threshold cycle; SARS-CoV, severe acute respiratory syndrome–associated coronavirus; RT-PCR, reverse transcription–polymerase chain reaction; GE, genome equivalents/assay; pp1ab, nonstructural polyprotein 1ab gene; n.d., not determined. ^b^See Table 1.

**Figure:**
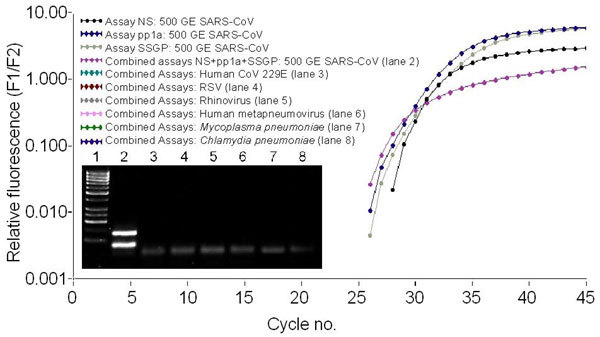
LightCycler amplification curves of the three single SARS-coronavirus (CoV)-specific assays and the combined assay. Five hundred genome equivalents (GE) of SARS-CoV were amplified with the three single assays-NS pp1a, NS pp1ab, and SS GP-as well as with the combination of the three assays. No fluorescence signal was observed for control pathogens. In the inset, the respective agarose gel analysis of the amplification products in a 3% gel is shown. Lane 1: 50-bp marker, lane 2: 500 GE SARS-CoV amplified with the assay combination (products of assays NS pp1ab and SS GP are not clearly distinguishable because of sizes of 88 bp and 79 bp, respectively), lane 3: human CoV 229E, lane 4: respiratory syncytial virus, lane 5: rhinovirus, lane 6: human metapneumovirus, lane 7: Mycoplasma pneumoniae, or lane 8: Chlamydia pneumoniae.

Using the single or combined assays, we analyzed 27 bronchoalveolar-lavage fluid samples from 19 suspected SARS case-patients and 8 probable SARS patients (according to the Robert Koch-Institut case definition, available from http://www.rki.de/INFEKT/SARS/AOLG-FALLDEF-ARSUU.PDF). All samples were positive for L13 and cyclophilin control sequences when amplified in parallel. In agreement with the previously published assay results (5), SARS-CoV was detectable in three samples from eight probable SARS patients, without explicit differences in the C_T_ value of individual assays when the single or combined assays were used. These patients were seropositive and are regarded as confirmed SARS patients. Respiratory samples and stool samples taken 8 days later from the remaining 5 probable patients as well as the 19 persons with suspected disease were negative by RT-PCR. Moreover, these patients remained seronegative and are regarded as unconfirmed SARS patients. In addition, 35 serum samples from patients with SARS-CoV infection obtained 1–52 days after disease onset were analyzed. Between 20 and 1,000 GE/mL of SARS-CoV-specific RNA was detected in 21 of 35 serum samples, even when serum was obtained from patients 1 day after disease onset. (A detailed description of this study will be published later.)

## Conclusions

None of the assays displayed cross-reactivity to clinical samples containing human cDNA from blood; human CoV 229E; influenza viruses A and B; parainfluenzaviruses 1, 2, and 3; respiratory syncytial virus; rhinoviruses; enteroviruses; adenoviruses 1–10; human metapneumovirus; *Mycoplasma pneumoniae*; or *Chlamydia pneumoniae*. For these pathogens, we obtained neither a fluorescent signal nor an amplification product in subsequent agarose gel analysis (selection shown in Figure). Although we focused on a one-step RT-PCR to decrease handling and total assay time, the three real-time RT-PCR assays can also be performed as two-step RT-PCR, including a separate cDNA synthesis step followed by PCR, and then finally using appropriate ready-to-use master mixes and the same cycling conditions, omitting the RT step.

The single assays and the combined assay were also used in an external quality assessment to detect SARS-CoV organized by the ENIVD. All assays could detect SARS-CoV in 7 of 11 samples with virus loads ranging from 5x10^6^ to 2x10^3^ GE of two isolates of SARS-CoV per milliliter sample without false-positive or false-negative results. While the application of three single assays to detect SARS-CoV leads to a higher reliability of negative results, reflecting the negative outcome of three independent amplification reactions, it is a more expensive approach than combining the assays.

In conclusion, the real-time RT-PCR assays we describe provide a fast and reliable tool that can complement and improve recently introduced techniques for SARS diagnostics. Parallel amplification of two human reference genes, L13 and cyclophilin, confirmed negative results in clinical samples by demonstrating amplifiable RNA. The separation of the control reaction was chosen to guarantee the high sensitivity of the SARS-CoV detection of <10 GE of SARS-CoV per reaction. An RT-PCR run is completed in <1 h, depending on the real-time PCR platform. In cases of small amounts of material or in an emergency situation with a high throughput of samples, the three SARS-CoV–specific assays can be combined into one RT-PCR reaction without loss of sensitivity. Furthermore, as the ambiguous diagnostic results in a hospital in Canada have recently shown (17), targeting three different regions distributed over the whole genome considerably reduces the risk for false-negative results caused by virus sequence modifications.
